# Notification that new names of prokaryotes, new combinations and new taxonomic opinions have appeared in volume 74, part 12 of the IJSEM

**DOI:** 10.1099/ijsem.0.006650

**Published:** 2025-03-28

**Authors:** Aharon Oren, Markus Göker

**Affiliations:** 1 The Institute of Life Sciences, The Hebrew University of Jerusalem, The Edmond J. Safra Campus, 9190401 Jerusalem, Israel; 2 Leibniz Institute DSMZ – German Collection of Microorganisms and Cell Cultures, Inhoffenstrasse 7B, 38124 Braunschweig, Germany

This listing of names of prokaryotes published in a previous issue of the *IJSEM* is provided as a service to microbiology to assist in the recognition of new names and new combinations. This procedure was proposed by the Judicial Commission [1]. The names given herein are listed according to the Rules of priority (i.e., the time and date of publication of the articles on the journal’s platform and the order of valid publication of names in the original articles) [2].

**Table:** 

Order of article publication	Name/author	Proposed as	Article no.	Page no.
1	*Sanguibacter biliveldensis* Kiel *et al*. 2024	sp. nov.	006560	7
2	*Pseudomonas boreofloridensis* Fullem *et al*. 2024	sp. nov.	006596	6
2	*Pseudomonas citrulli* Fullem *et al*. 2024	sp. nov.	006596	7
3	*Dictyobacter halimunensis* Rachmania *et al*. 2024	sp. nov.	006600	8
4	*Stenotrophomonas beteli* (Ragunathan 1928) Nguyen *et al*. 2024	comb. nov. (basonym: ‘*Bacterium betle’* Ragunathan 1928)	006602	15
4	*Stenotrophomonas forensis* Nguyen *et al*. 2024	sp. nov.	006602	15
4	*Stenotrophomonas hibiscicola* (Moniz 1963) Nguyen *et al*. 2024	comb. nov. [basonym: *Pseudomonas hibiscicola* Moniz 1963 (Approved Lists 1980)]	006602	15
5	*Bifidobacterium apicola* Wang *et al*. 2024	sp. nov.	006599	7
6	*Methanochimaera* Zhou *et al*. 2024	gen. nov.	006593	7
6	*Methanochimaera problematica* Zhou *et al*. 2024	sp. nov.	006593	8
6	*Methanoeremita* Zhou *et al*. 2024∗	gen. nov.	006593	8
6	*Methanoeremita antiquus* (Mochimaru *et al*. 2016) Zhou *et al*. 2024∗	comb. nov. (basonym: *Methanomicrobium antiquum* Mochimaru *et al*. 2016)	006593	8
7	*Streptomyces cavernicola* Chamroensaksri *et al*. 2024	sp. nov.	006563	7
7	*Streptomyces solicavernae* Chamroensaksri *et al*. 2024	sp. nov.	006563	9
7	*Streptomyces luteolus* Chamroensaksri *et al*. 2024	sp. nov.	006563	9
8	*Amycolatopsis heterodermiae* Somphong *et al*. 2024	sp. nov.	006598	7
8	*Actinacidiphila polyblastidii* Somphong *et al*. 2024	sp. nov.	006598	8
9	*Herbaspirillum huttiense* subsp. *lycopersici* Poudel *et al*. 2024	subsp. nov.	006597	8
9	*Herbaspirillum huttiense* subsp. *nephrolepidis* Poudel *et al*. 2024	subsp. nov.	006597	8
10	*Geodermatophilus maliterrae* Ben Tekaya *et al*. 2024	sp. nov.	006603	6
11	*Bacteriovorax antarcticus* Mun *et al*. 2024	sp. nov.	006607	11
12	*Sphingomonas longa* Kim *et al*. 2024	sp. nov.	006572	8
12	*Sphingomonas mollis* Kim *et al*. 2024	sp. nov.	006572	8
12	*Sphingomonas aurea* Kim *et al*. 2024	sp. nov.	006572	8
13	*Segatella asaccharophila* Makiura *et al*. 2024	sp. nov.	006606	7
14	*Bengtsoniella* Pardesi *et al*. 2024	gen. nov.	006615	5
14	*Bengtsoniella intestinalis* Pardesi *et al*. 2024	sp. nov.	006615	7
15	*Actinomadura monticuli* Lee *et al*. 2024	sp. nov.	006609	10
16	*Sphingobacterium siyangense* subsp. *siyangense* (Liu *et al*. 2008) Li *et al*. 2024	comb. nov. (basonym: *Sphingobacterium siyangense* Liu *et al*. 2008)	006610	9
16	*Sphingobacterium siyangense* subsp. *cladoniae* (Lee *et al*. 2013) Li *et al*. 2024	comb. nov. (basonym: *Sphingobacterium cladoniae* Lee *et al*. 2013)	006610	9
17	*Pseudomonas serbiensis* Fullem *et al*. 2024	sp. nov.	006613	7

*Not validly published. Culture collection numbers for the type species were given in the abstract but not in the protologue; the protologue does not list the properties of the taxon and does not refer to a previously published description [Rule 27(2)].

The following taxonomic opinions (i.e., the emendation of circumscriptions or the creation of synonyms) do not affect the status of a name under the International Code of Nomenclature of Prokaryotes. These taxonomic opinions can neither be considered approved nor disapproved by the International Committee on Systematics of Prokaryotes.

**Table:** 

Name/author	Article no.	Page no.
*Actinomadura glauciflava* Lu *et al*. 2003 pro synon. *Actinomadura luteofluorescens* (Shinobu 1962) Preobrazhenskaya *et al*. 1975 (Approved Lists 1980)	006609	10
*Actinomadura luteofluorescens* (Shinobu 1962) Preobrazhenskaya *et al*. 1975 (Approved Lists 1980) emend. Lee *et al*. 2024	006609	10
*Sphingobacterium soli* Fu *et al*. 2017 pro synon. *Sphingobacterium cellulitidis* Huys *et al*. 2017	006610	8
*Sphingobacterium cellulitidis* Huys *et al*. 2017 emend. Li *et al*. 2024	006610	8
